# Carbetocin is a Functional Selective Gq Agonist That Does Not Promote Oxytocin Receptor Recycling After Inducing β‐Arrestin‐Independent Internalisation

**DOI:** 10.1111/jne.12363

**Published:** 2016-04-25

**Authors:** I. Passoni, M. Leonzino, V. Gigliucci, B. Chini, M. Busnelli

**Affiliations:** ^1^ Department of Medical Biotechnology and Translational MedicineUniversità degli Studi di MilanoMilanItaly; ^2^ CNRInstitute of NeuroscienceMilanItaly; ^3^ Humanitas Clinical and Research InstituteRozzanoItaly

**Keywords:** carbetocin, oxytocin receptor, vasopressin receptor, β‐arrestins, receptor recycling

## Abstract

Carbetocin, a long‐acting oxytocin analogue, has been reported to elicit interesting and peculiar behavioural effects. The present study investigated the molecular pharmacology of carbetocin, aiming to better understand the molecular basis of its action in the brain. Using bioluminescence resonance energy transfer biosensors, we characterised the effects of carbetocin on the three human oxytocin/vasopressin receptors expressed in the nervous system: the oxytocin receptor (OXTR) and the vasopressin V1a (V1aR) and V1b (V1bR) receptors. Our results indicate that (i) carbetocin activates the OXTR but not the V1aR and V1bR at which it may act as an antagonist; (ii) carbetocin selectively activates only the OXTR/Gq pathway displaying a strong functional selectivity; (iii) carbetocin is a partial agonist at the OXTR/Gq coupling; (iv) carbetocin promotes OXTR internalisation via a previously unreported β‐arrestin‐independent pathway; and (v) carbetocin does not induce OXTR recycling to the plasma membrane. Altogether, these molecular pharmacology features identify carbetocin as a substantially different analogue compared to the endogenous oxytocin and, consequently, carbetocin is not expected to mimic oxytocin in the brain. Whether these unique features of carbetocin could be exploited therapeutically remains to be established.

Oxytocin is a small nonapeptide produced by magnocellular and parvocellular neurones of the supraoptic and paraventricular nuclei of the hypothalamus; from these sites of synthesis, the peptide is delivered to the periphery by axons projecting to the posterior pituitary and to the central nervous system (CNS) by dendrites and axonal collaterals 1, 2. In the periphery, oxytocin has several functions, including the contraction of uterine smooth muscles during labour, an effect that has been extensively exploited for decades to promote contractions during the third stage of labour and to control bleeding after childbirth 3, 4. Oxytocin also promotes the contraction of the mammary myoepithelium during lactation 5 and the intranasal use of oxytocin to stimulate lactation in breastfeeding women was approved by the Food and Drug Administration in the USA in 1960, even though it was discontinued in 1997 for commercial reasons.

In the CNS, oxytocin acts as a neurotransmitter/neuromodulator regulating several aspects of social behaviour, learning and memory processes, and stress and anxiety responses 6, 7. On the basis of its capability to promote social interactions in all vertebrates, including humans, oxytocin has been proposed as a clinical treatment for relieving social impairments associated with neurodevelopmental and psychiatric disorders 8, 9. In the past decade, a number of clinical trials have been performed in autism and schizophrenia but, unfortunately, no consensus on the real efficacy of oxytocin on these conditions has yet been reached 10. Several factors contribute to the limited/controversial efficacy of oxytocin in the clinical practice: (i) oxytocin has a short half‐life in the plasma 11 and in the cerebrospinal fluid 12 and, consequently, its pharmacological effects are short lived; (ii) oxytocin does not cross the blood–brain barrier in a significant amount 12 and thus, when given by parenteral administration, it does not reach the brain; (iii) intranasal oxytocin administration, considered as a way of delivering exogenous oxytocin directly into the brain, has not yet been proven to do so 13, leading to very poorly defined pharmacokinetics parameters (doses, intervals, metabolites); (iv) oxytocin binds to and activates the vasopressin V1a and V1b receptor subtypes, which are also highly expressed in the brain where they promote effects different from (and even the opposite to) those of the oxytocin receptor (OXTR) 14; and (v) oxytocin promotes OXTR coupling to a number of different G‐protein subtypes and β‐arrestins 15, 16, leading to the activation of multiple signalling pathways, whose precise roles within the brain are currently unknown.

To fully exploit the pharmacological potential of the OXTR and, at the same time, to overcome the limits of endogenous oxytocin, a number of oxytocin analogues have been developed 17. In particular, in an attempt to increase the half‐life of the hormone, the deamino‐1‐monocarba‐(2‐O‐methyltyrosine)‐oxytocin (carbetocin) was synthesised by deaminating the N‐terminus and by replacing the disulphide (S‐S) bridge between Cys 1‐6 with a CH_2_‐S bond that connects a butyric acid functional group, at the N‐terminus, and Cys 5. These modifications resulted in the very effective protection of carbetocin from aminopeptidase degradation and disulphidase cleavage 18, thus increasing the half‐life of this oxytocin analogue in the peripheral circulation (carbetocin 85–100 min versus oxytocin 3.4 min) 11, 19. This increase in half‐life was accompanied by an increased efficacy *in vivo*, as demonstrated by animal studies in which carbetocin was able to induce prolonged uterine contraction 20 and milk let‐down activity 21. Similarly, in humans, carbetocin had a prolonged effect on post‐partum uterine activity in terms of both a higher amplitude and frequency of contractions compared to oxytocin 22. Concerning the central effects of carbetocin, studies in rodents demonstrated that carbetocin reduced anxiety‐like behaviours in the elevated plus maze test 23, had antidepressant‐like effects in the forced swimming test 24 and attenuated the negative emotional consequences of opioid withdrawal 25, 26.

Interestingly, behavioural studies directly comparing carbetocin and oxytocin have reported different (and in some cases opposite) effects. By contrast to oxytocin, carbetocin failed to revert deficits in pre‐pulse inhibition (PPI) of the startle reflex in Brown Norway rats, a rat strain that has naturally low PPI, thus suggesting that carbetocin does not have antipsychotic‐like central effects 27. In the open field paradigm, although oxytocin induced a reduction in exploratory activity and increased grooming time, carbetocin had a slightly increased effect on exploratory activity and no effect on grooming 28. Moreover, in rats previously exposed to restraint stress, oxytocin reduced locomotion and increased grooming, whereas carbetocin increased locomotion and decreased grooming 29. Finally, carbetocin (but not oxytocin) had long‐term ameliorating effects on restraint stress‐induced behavioural changes 30.

The present study investigated in detail the pharmacological properties of carbetocin on the OXTR and vasopressin V1a and V1b receptors, aiming to identify the molecular mechanisms that could account for the differences observed between carbetocin and oxytocin at the behavioural level. We found that carbetocin is highly selective for the OXTR compared to the vasopressin V1a and V1b receptors, and that it specifically activates only the OXTR/Gq pathway, displaying functional selective properties. Finally, we investigated the effects of carbetocin on OXTR internalisation and intracellular trafficking and found that carbetocin promotes OXTR internalisation through a previously undescribed β‐arrestin‐independent internalisation pathway, which also negatively influences OXTR recycling.

## Materials and methods

### Peptide and reagents

Oxytocin, carbetocin and arginine vasopressin were obtained from Bachem (Bubendorf, Switzerland). Coelenterazine h was obtained from Molecular Probes, Invitrogen (Carlsbad, CA, USA) and coelenterazine 400a (CLz400) was from Biotium (Hayward, CA, USA).

### Cell culture

HEK293 cells were maintained in Dulbecco's modified Eagle's medium (DMEM) supplemented with 10% foetal bovine serum (FBS), 200 U/ml penicillin, 200 mg/ml streptomycin and 2 mm l‐glutamine (all purchased from Sigma‐Aldrich, St Loiuis, MO, USA).

### cDNA constructs

The expression vectors for G‐proteins fused to Renilla luciferase Gα_q_‐97‐Rluc8, Gα_i1_‐91‐Rluc8, Gα_i2_‐91‐Rluc8, Gα_i3_‐91‐Rluc8 and Gα_o_‐91‐Rluc8 and the vectors for GFP10‐Gγ_2_ and Gβ_1_ were a gift from Dr Celine Gales (Inserm U858, Toulouse, France) and have been described previously 31. The plasmids encoding for the human OXTR (hOXTR) and the hOXTR‐Rluc vectors were also the same as reported previously 15. The plasmids for V1aR and V1bR were a gift from Professor G. Guillon and Dr B. Mouillac (IGF, CNRS, Montpellier, France). The expression vectors for β‐arrestins‐Venus were kindly provided by Professor M. Locati (Humanitas Research Hospital Rozzano, Italy). The hOXTR‐RFP construct was generated amplifying the entire coding sequence of hOXTR by a polymerase chain reaction (PCR) and using the forward primer 5′‐CAAACTCGAGATGGAGGGCGCGCTCGCAG‐3′ and the reverse primer 5′‐GTTTGGATCCGCCGTGGATGGCTGGGAG‐3′ and the Pfu DNA polymerase (Promega, Madison, WI, USA). The resulting PCR product was subcloned into the Tag‐RFP plasmid (Evrogen, Moscow, Russia) using the *Xho*I and *Bam*HI restriction sites. hOXTR‐RFP fusion constructs were sequenced on both DNA strands (MWG Eurofins, Ebersberg, Germany).

### Transfections

For bioluminescence resonance energy transfer (BRET) experiments, cells were seeded at a density of 3.1 × 10^6^ cells/well in 100‐mm plates and transfected after 24 h with polyethyleneimine (PEI linear, relative molecular mass of 25 000; Polysciences, Inc., Warrington, PA, USA) as described previously 15. Twenty‐four hours after transfection, the supplemented DMEM was renewed, and the cells were cultured for a further 24 h before the experiments. Forty‐eight hours after transfection, the cells were washed twice, harvested and resuspended with phosphate‐buffered saline (PBS) supplemented with 0.1% (w/v) glucose at room temperature.

For imaging experiments, cells were seeded on glass coverslips (3 × 10^5^ cells/coverslip), allowed to grow for 24 h (60% confluence) and transfected with TurboFect (Thermo Scientific). For each transfection sample, 1 μg of cDNA was mixed with 3 μl of TurboFect in 400 μl of DMEM supplemented with 2 mm l‐glutamine and incubated for 15 min at room temperature. DNA/TurboFect complexes were gently added to the cells in 3.6 ml of complete medium supplemented with 10% FBS. Twenty‐four hours after transfection, the supplemented DMEM was renewed and the cells were cultured for a further 24 h before the experiments.

### BRET assays

To screen for the effects of the different ligands on G‐protein activation, we performed BRET^2^ assays. Plasmids encoding for GFP10‐Gγ_2_, Gβ_1_ and the hOXTR or vasopressin receptors (V1aR and V1bR) were co‐transfected together with the different Gα‐Rluc8 plasmids in HEK293 cells. To study OXTR–β‐arrestin interactions, we used kinetic BRET^1^ experiments and the cells were co‐transfected with hOXTR‐Rluc and β‐arrestin1‐Venus or β‐arrestin2‐Venus. Forty‐eight hours after transfection, cells were washed twice, harvested and resuspended in PBS‐glucose 0.1% (w/v) at room temperature. The protein content of cells was determined using the DC Assay (Bio‐Rad, Hercules, CA, USA) and cells were resuspended to the final protein concentration of 1 mg/ml. Cells (80 μg of proteins/well) were then distributed in a white 96‐well microplate (Optiplate; PerkinElmer Life Sciences, Boston, MA, USA). For the G‐protein activation studies, cells were incubated for 2 min with the indicated ligands or PBS (untreated cells) before substrate addition. BRET^2^ energy transfer between Rluc8 and GFP10 was measured immediately after the addition of the Rluc8 substrate coelenterazine 400a (5 μm; Biotium), using an Infinite F500 reader plate (Tecan, Männedorf, Switzerland) that allows the sequential integration of light signals detected with two filter settings (Rluc8 filter, 370–450 nm; GFP10 filter, 510–540 nm). The data were recorded and the BRET^2^ signal was calculated as the ratio between GFP10 emission and the light emitted by Rluc8. The changes in BRET signal induced by the ligands were expressed on graphs as the ‘BRET ligand effect’ using the formula

$$\text{BRET ligand effect}=\left(\text{emission GFP}10_{\mathrm{ligand}}/\text{emission Rluc}8_{\mathrm{ligand}}\right)-\left(\text{emission GFP}10_{\mathrm{PBS}}/\text{emission Rluc}8_{\mathrm{PBS}}\right).$$

To study the kinetics of the OXTR–β‐arrestin interactions, coelenterazine h (the substrate specific for BRET^1^ experiments; Molecular Probes, Invitrogen) was added to cells 8 min before the addition of oxytocin (10 μm) or carbetocin (10 μm) and readings were registered using an Infinite F500 reader plate (Tecan) and filter set (Rluc filter, 370–480 nm; Venus filter, 520–570 nm). To determine the half‐time (t_1⁄2_) of oxytocin‐ and carbetocin‐induced BRET, the data were recorded as the difference between the ‘ligand‐promoted BRET’ signal and the average of the baseline (PBS‐treated) BRET signal, and the time at which the half‐BRET peak was reached was estimated.

### Fluorescence microscopy, β‐arrestin recruitment, internalisation and recycling studies

In β‐arrestin recruitment studies, cells were co‐transfected with hOXTR‐RFP and β‐arrestin1‐ or β‐arrestin2‐Venus constructs and, after 48 h, were preincubated for 30 min in serum‐free medium at 37 °C and treated with oxytocin (100 nm) or carbetocin (1 μm) for 2 min. In the internalisation and recycling studies, cells were transfected with hOXTR‐RFP. Forty‐eight hours after transfections, cells were preincubated for 30 min in serum‐free medium at 37 °C and treated with oxytocin (100 nm) or carbetocin (1 μm) for 15 min. In the recycling experiments, agonist was removed after a period of 15 min with an acid wash (pH 3.3) (150 mm NaCl, 5 mm CH_3_COOH) and the cells were then incubated for 4 h in serum‐free medium. All the cellular processes were blocked by placing the dishes on ice and cells were immediately processed for fluorescence microscopy analysis. Cells seeded on glass coverslips were washed twice with 10 mm sodium phosphate buffer (pH 7.4), containing 150 mm NaCl [high‐salt buffer (HS)] and fixed for 20 min at room temperature with 4% (w/v) paraformaldehyde. Fixed cells were stained with 4′,6‐diamidino‐2‐phenylindole, washed five times with HS, and then once with H_2_O. Glass coverslips were mounted on glass slides with Mowiol (Sigma‐Aldrich) and analysed using an Axiovert 200M (Carl Zeiss, Oberkochen, Germany) confocal system equipped with a spinning disc (PerkinElmer Life Sciences) with a × 63 objective.

### Statistical analysis

All data were analysed using prism, version 5.0 (GraphPad Software Inc., San Diego, CA, USA). BRET data are all expressed as the mean ± SEM, calculated by simultaneous analysis of a minimum of three different experiments performed at least in duplicate. Ligand‐induced BRET ratios were analysed with one‐way anova followed by Dunnett's post‐hoc test, comparing oxytocin, carbetocin or arginine vasopressin with vehicle within each G‐protein subtype, aiming to determine statistically significant differences in treatments (*P* *<* *0.05, **P* *<* *0.01, ***P* *<* *0.001). Concentration–response BRET curves were analysed by means of nonlinear curve fitting using the sigmoidal dose–response equation. BRET kinetics data were normalised by setting the zero time point immediately after the addition of the ligand, and the data were analysed by means of nonlinear least‐squares fitting to the one‐phase exponential association equation.

## Results

### Carbetocin, in contrast to oxytocin, is a ‘functional selective’ OXTR/Gq agonist

We have previously demonstrated that the native peptide oxytocin can activate OXTR coupling to different G‐protein subtypes: Gq, Gi and Go 15.

**Figure 1:**
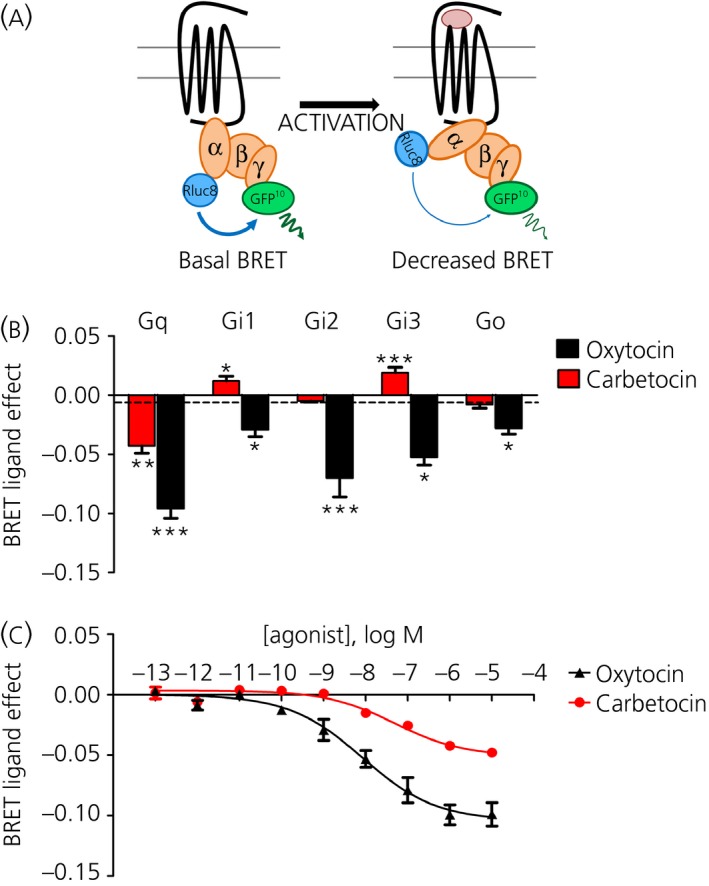
Carbetocin selectively activates oxytocin receptor (OXTR)/Gq coupling with partial efficacy. (a) Schematic representation of bioluminescence resonance energy transfer (BRET) between Rluc8 (the donor) and GFP10 (the acceptor), introduced into the α helical domain of the Gα subunits and the N‐terminal domain of Gγ_2_ (GFP10‐Gγ_2_), respectively. Agonist‐induced OXTR‐Gα activation leads to a conformational rearrangement of the heterotrimeric G‐protein complex that corresponds to a decrease in the BRET ratio. (b) BRET was measured in HEK293 cells co‐expressing hOXTR, GFP10‐Gγ_2_, Gβ_1_ and different Rluc8‐tagged Gα subunits: Gq, Gi1, Gi2, Gi3, Go. The results represent the agonist‐promoted BRET signal after oxytocin (10 μm) or carbetocin (10 μm) and are expressed as the mean ± SEM. Statistical differences between agonist‐promoted BRET in the presence of the indicated Gα proteins and PBS‐treated controls (dotted line) were determined by one‐way anova followed by Dunnett's post‐hoc test (*P* *<* *0.05, **P* *<* *0.01, ***P* *<* *0.001). (c) Cells were transfected with hOXTR, GFP10‐Gγ_2_, Gβ_1_, Gαq‐Rluc8 and were stimulated with different concentrations of oxytocin or carbetocin (from 10^−13^ to 10^−5^ m) for 2 min. The results represent the ‘BRET ligand effect’ signals for oxytocin and carbetocin and are expressed as the mean ± SEM of a minimum of three independent experiments, each performed at least in duplicate.

To investigate the coupling specificity of human OXTR (hOXTR) in response to carbetocin, we employed BRET‐based biosensors (Fig. 1 a). HEK293 cells were transfected with the hOXTR in combination with different Gα subunits fused to the BRET energy donor *Renilla reniformis* luciferase (Gα‐Rluc8), and with the energy acceptor, a blue‐shifted variant of *Aequorea Victoria* green fluorescent protein (GFP10), N‐terminally fused to the Gγ_2_ subunit (GFP10‐Gγ_2_) 31. Ligand‐induced OXTR activation of the G‐protein leads to GDP release coupled to a conformational rearrangement of the heterotrimeric complex and, consequently, a decrease in the BRET ratio (Fig. 1 a). To screen oxytocin and carbetocin for their efficacy to activate OXTR signalling, we used a 10 μm dose, which, for oxytocin, is known to maximally activate all the different G‐protein subtypes 15 and, for carbetocin, is 1000‐fold greater than its affinity for the OXTR.

For all the G‐proteins analysed, one‐way anova showed an effect of treatment on OXTR: Gq (F_2,51_ = 52.57, P < 0.0001); Gi1 (F_2,17_ = 10.53, P < 0.01); Gi2 (F_2,18_ = 204.3, P < 0.0001); Gi3 (F_2,17_ = 34.55, P < 0.0001); and Go (F_2,40_ = 17.21, P < 0.0001). As demonstrated previously 15, oxytocin (10 μm) activated Gq, Gi1, Gi2, Gi3 and Go proteins; by contrast, carbetocin (10 μm) activated only the Gq protein, indicating that carbetocin is a OXTR/Gq ‘functional selective’ analogue (Fig. 1 b). Notably, for carbetocin, we observed a modest but significant increase in the energy transfer in the presence of Gi1 and Gi3 (Fig. 1 b), indicating a particular rearrangement of the receptor associated with a closer organisation of the trimeric G‐protein complex, which could indicate inverse agonism 32.

### Carbetocin is a partial agonist for the OXTR/Gq coupling

Using the same Gq BRET biosensor (Fig. 1 a), we obtained dose–response curves. We found an increased EC_50_ for carbetocin: EC_50_ = 48.8 ± 16.09 nm for carbetocin and EC_50_ = 9.7 ± 4.43 nm for oxytocin, respectively (Fig. 1 c). This right‐shifted EC_50_ is congruent with the reported affinity for the OXTR (K_i_ = 7 nm), which is approximately 10‐fold lower than that of oxytocin (K_i_ = 0.71 nm). We also found that maximal activation (BRET_max_) induced by carbetocin was approximately half (45 ± 6%) that of oxytocin, suggesting that carbetocin is a partial OXTR/Gq agonist (Fig. 1 c). This is in agreement with earlier observations 33, 34 showing that carbetocin on isolated uterine strips generates a reduced maximal contractile response compared to oxytocin, indicating a ‘weaker’ activation of the OXTR.

### Carbetocin does not activate vasopressin V1a and V1b receptors and acts as a competitive antagonist

A previous study has reported that carbetocin is able to bind to vasopressin receptors in rat myometrial homogenates 34; therefore, we tested the capability of carbetocin with respect to activating human V1a and V1b receptors with the same BRET biosensors used for OXTR (Fig. 1 a).

For all of the G‐proteins analysed, one‐way anova showed an effect of treatment on V1aR: Gq (F_3,33_ = 1387, P < 0.0001); Gi1 (F_3,36_ = 86.23, P < 0.0001); Gi2 (F_3,33_ = 129.4, P < 0.0001); Gi3 (F_3,39_ = 195.6, P < 0.0001); and Go (F_3,28_ = 11.02, P < 0.0001). An effect of treatment was observed also for V1bR: Gq (F_3,24_ = 358.5, P < 0.0001); Gi1 (F_3,24_ = 30.46, P < 0.0001); Gi2 (F_3,24_ = 72.92, P < 0.0001); Gi3 (F_3,24_ = 95.75, P < 0.0001); and Go (F_3,24_ = 22.27, P < 0.001).

**Figure 2:**
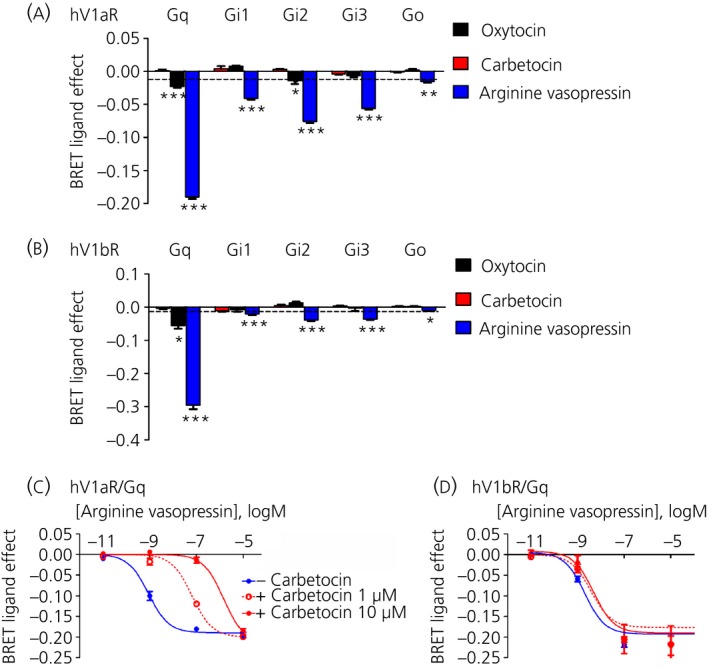
Carbetocin does not activate and acts as competitive antagonist for vasopressin V1a (V1aR) and vasopressin V1b (V1bR) receptors. (a) V1aR or (b) V1bR were transfected together with GFP10‐Gγ_2_, Gβ_1_ and different Rluc8‐tagged Gα subunits: Gq, Gi1, Gi2, Gi3, Go. The results represent the agonist‐induced bioluminescence resonance energy transfer (BRET) signal with oxytocin (10 μm), carbetocin (10 μm) or arginine vasopressin (10 μm) and are expressed as the mean ± SEM of a minimum of three independent experiments, each performed at least in duplicate. Statistical differences between ‘BRET ligand effect’ signal in the presence of the indicated Gα proteins and phosphate‐buffered saline‐treated controls (dotted line) were determined by one‐way anova followed by Dunnett's post‐hoc test (*P* *<* *0.05, **P* *<* *0.01,***P* *<* *0.001). Cells transfected with Gαq‐Rluc8, GFP10‐Gγ_2_, Gβ_1_ and (c) V1aR or (d) V1bR were stimulated with increasing concentrations of arginine vasopressin alone (blue line) or simultaneously treated with carbetocin 1 μm (red dotted line) or 10 μm (red solid line) for 2 min. The results represent the ‘BRET ligand effect’ signals for arginine vasopressin and arginine vasopressin + carbetocin and are expressed as the mean ± SEM of a minimum of three independent experiments, each performed at least in duplicate.

Even when used at a high concentration (10 μm) at least 500‐fold greater than its affinity for the vasopressin receptors 34, carbetocin was completely inactive on vasopressin V1aR and V1bR (Fig. 2 a,b). By contrast, the same high concentration (10 μm) of oxytocin selectively promoted the activation of V1aR/Gq and V1aR/Gi2, as well as V1bR/Gq. Moreover, the neuropeptide arginine vasopressin significantly promoted the coupling/activation of the vasopressin V1aR and V1bR to all G‐protein subtypes. Interestingly, our data for V1bR confirmed the results of the previous study 35 that used an *in vitro* luciferase‐based transcription reporter gene assay and reported no activation of this receptor by carbetocin at a concentration up to 10 μm. However, in contrast to the present study, a partial agonism for V1aR, arbitrarily assigned to < 70%, was reported in the previous study 35. Unfortunately, we cannot make direct comparisons between our BRET studies and the previous studies because, in the latter case, the nature of the responsive elements present in the promoter of their construct was not described, and we cannot therefore establish with certainty which signalling pathway was activated by the peptide.

We then tested the possibility that carbetocin acts as a competitive antagonist on the vasopressin V1a (Fig. 2 c) and V1b receptors (Fig. 2 d). Accordingly, we obtained dose–response curves for the activation of Gq in the presence of arginine vasopressin and carbetocin. Our results indicated that the simultaneous incubation with carbetocin resulted in a rightward shift of the concentration–response curves of arginine vasopressin without a noticeable change of the maximal response (Fig. 2 c,d). Moreover, the right‐shift of the arginine vasopressin curve is dose‐dependent, indicating that carbetocin behaves as a competitive antagonist on V1aR and V1bR.

### Carbetocin induces OXTR internalisation without recruiting β‐arrestins

Exposure to oxytocin leads to desensitisation of the OXTR 36. Desensitisation, which protects cells from overstimulation after prolonged agonist exposure, can occour very rapidly, within seconds or minutes, and is observed in the majority of G protein‐coupled receptors (GPCRs). It is a multistep phenomenon, in which the ability to respond to stimuli is switched off for varying lengths of time. The first step is the phosphorylation of the receptor, which inhibits G‐protein activation, followed by the binding of specific proteins called β‐arrestins that prevent G‐protein activation and promote receptor internalisation 37.

**Figure 3:**
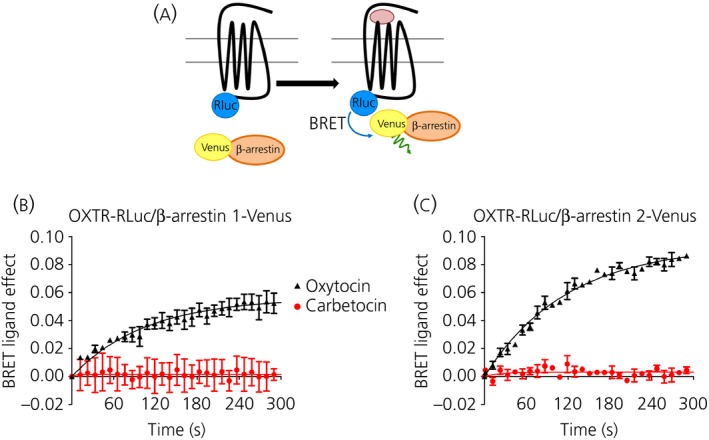
Carbetocin does not promote the recruitment of β‐arrestins. (a) Schematic representation of bioluminescence resonance energy transfer (BRET) between Rluc and Venus introduced at the C‐terminus of human oxytocin receptor (OXTR) (hOXTR) (hOXTR‐RLuc) and of the β‐arrestins (β‐arrestin‐Venus), respectively. HEK293 cells co‐expressing hOXTR‐Rluc and (b) β‐arrestin1‐Venus or (c) β‐arrestin2‐Venus were stimulated by maximal doses (10 μm) of oxytocin and carbetocin. BRET measurements were made in real‐time for 5 min. The results represent the ‘BRET ligand effect’ signal for oxytocin and carbetocin and are expressed as the mean ± SEM of a representative experiment (from three independent experiments) performed in duplicate. The curve was generated as a one‐phase association.

To determine whether carbetocin induced the recruitment of β‐arrestins, we used a ‘real‐time’ kinetic BRET assay in which the hOXTR‐RLuc construct acted as the energy donor, and the yellow variant of GFP (Venus) fused to the C‐terminus of β‐arrestin1 and β‐arrestin2 (β‐arrestin1‐Venus and β‐arrestin2‐Venus) acted as the acceptor (Fig. 3 a). In cells co‐expressing hOXTR‐Rluc and β‐arrestin1‐Venus, oxytocin at a final concentration of 10 μm (which we previously determined to be able to promote maximal β‐arrestin1 and β‐arrestin2 recruitment) 15 increased the BRET ratio with a t_1⁄2_ of 119 ± 18 s; this increase remained stable for at least 5 min (Fig. 3 b), thus indicating a rapid and sustained agonist‐induced association between the OXTR and β‐arrestin1. Similar results were obtained using the β‐arrestin2‐Venus construct, with a t_1⁄2_ of 52 ± 8 s (Fig. 3 c). By contrast, no variations in the BRET signal were observed in the presence of a high dose of carbetocin (10 μm) (Fig. 3 b,c). These data indicate that carbetocin did not promote OXTR–β‐arrestin association.

**Figure 4:**
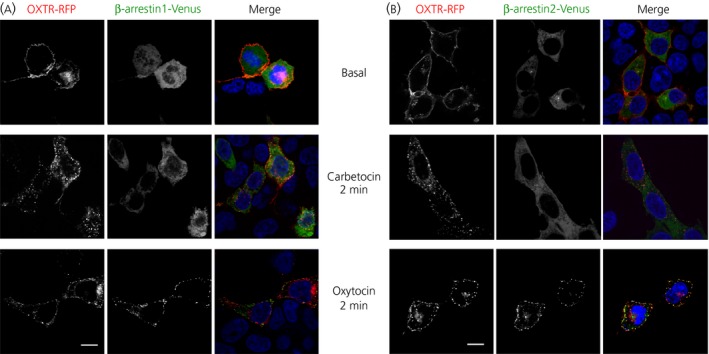
Carbetocin promotes oxytocin receptor (OXTR) internalisation independently of the recruitment of β‐arrestins. Representative confocal microscopy images of cells transfected with hOXTR‐RFP (red) and (a) β‐arrestin1‐Venus (green) and (b) β‐arrestin2‐Venus (green) treated with phosphate‐buffered saline (Basal), oxytocin (100 nm) or carbetocin (1 μm) for 2 min. After agonist stimulation, cells were fixed, incubated with 4′,6‐diamidino‐2‐phenylindole to stain nuclei and imaged by confocal microscopy. Images are representative of three independent experiments. Scale bar = 10 μm.

To determine whether the different recruitment of β‐arrestins affected OXTR internalisation, we used confocal microscopy studies. We used HEK293 cells transfected with hOXTR‐RFP and β‐arrestin1‐Venus (Fig. 4 a) or β‐arrestin2‐Venus (Fig. 4 b). In this experiment, we stimulated cells for 2 min with 100 nm oxytocin, a concentration that is 100‐fold greater than its K_i_ and EC_50_ for OXTR and that was also previously shown to induce the complete internalisation of OXTR without affecting its trafficking 36. Because carbetocin is characterised by K_i_ and EC_50_ values for OXTR that are 10‐fold bigger than those for oxytocin, we used 1 μm carbetocin for the same studies 38. As shown in Fig. 4, before agonist exposure (baseline) the hOXTR‐RFPs were localised at the plasma membrane, whereas there was a homogeneous distribution of β‐arrestins‐Venus in the cytoplasm. Incubation with oxytocin (but not carbetocin) induced β‐arrestins accumulation at the plasma membrane that positively colocalised with OXTR, confirming that oxytocin (but not carbetocin) promoted the recruitment of β‐arrestins. Moreover, in the presence of carbetocin and oxytocin, we observed fluorescent puncta distributed at the plasma membrane and in the cytosol, which presumably represented the OXTR internalised and localised in endocytic vesicles. Altogether, these results indicate that, in contrast to oxytocin, carbetocin promotes OXTR internalisation with a pathway independent of β‐arrestins.

### OXTR internalised upon carbetocin stimulation does not recycle to the plasma membrane and remains intracellular

**Figure 5:**
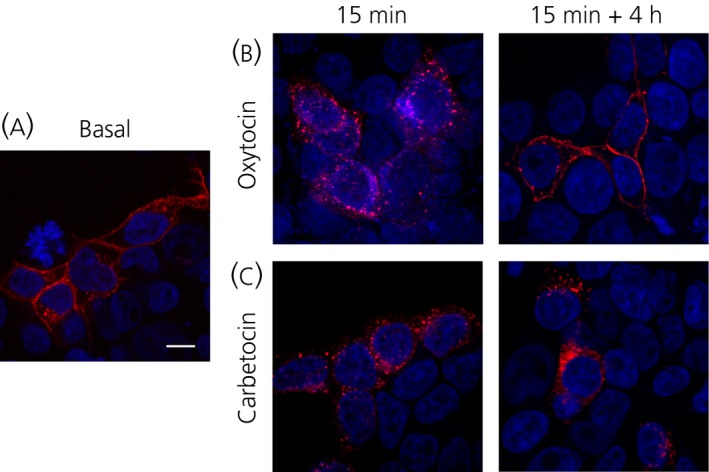
Carbetocin treatment prevents oxytocin receptor (OXTR) recycling to the plasma membrane after agonist removal. Representative confocal microscopy images of cells transfected with hOXTR‐RFP (red) (a) at basal conditions and (b) after 15 min oxytocin (100 nm) or (c) carbetocin (1 μm). In recycling experiments, cells were treated for 15 min with oxytocin (100 nm) or carbetocin (1 μm), washed in acidic buffer, and kept in the absence of agonists for 4 h. They were then fixed, incubated with 4′,6‐diamidino‐2‐phenylindole to stain nuclei and imaged by confocal microscopy. Images are representative of three independent experiments. Scale bar = 10 μm.

We have previously demonstrated that OXTRs endocytosed in response to 100 nm oxytocin are not sorted to the lysosomes for degradation and they are recycled back to the cell surface 36. We treated hOXTR‐RFP transfected HEK293 cells (Fig. 5 a, basal) with oxytocin (100 nm) (Fig. 5 b) or carbetocin (1 μm) (Fig. 5 c) for 15 min and we observed no fluorescence staining at the plasma membrane and a great number of intracellular fluorescent puncta, indicating that, at this time, both agonists promoted massive OXTR internalisation (Fig. 5 b,c). For recycling, we treated cells for 15 min, we removed the agonist with an acid wash, and we maintained cells at 37 °C for 4 h. In our experiments, we did not block protein synthesis because we have previously demonstrated that, after 4 h, the OXTR *de novo* synthesis does not play a major role in the reappearance of the receptor at the cell surface 36. With oxytocin, in agreement with our previous observations 36, the fluorescence staining reappeared at the plasma membrane after 4 h and was as intense as under basal conditions (Fig. 5 b). By contrast, after carbetocin, OXTRs remained in intracellular vesicles and did not recycle to the plasma membrane (Fig. 5 c).

These results indicate that carbetocin efficiently promotes OXTR internalisation in the absence of the recruitment of β‐arrestins, and this process is not coupled to OXTR recycling.

## Discussion

In the present study, we report the molecular pharmacology of carbetocin, an analogue of oxytocin originally designed to achieve a long half‐life 18. In particular, we investigated: (i) the coupling selectivity of carbetocin for the human OXTR; (ii) the agonistic properties of carbetocin for the human OXTR, V1a and V1b receptors; and (iii) the carbetocin‐induced OXTR internalisation and recycling. Our data indicate that carbetocin possesses specific and peculiar features in all these aspects, suggesting a pharmacological profile and therapeutic potential different from that of the endogenous neuropeptide oxytocin.

First, we investigated the coupling efficiency and selectivity of carbetocin for the human OXTR. Our data indicate that carbetocin is able to activate Gq and, in the case of Gi1 and Gi3, promotes an increase in the energy transfer, which could indicate inverse agonism 32, demonstrating a unique functional selective bias towards Gq.

In addition, compared to oxytocin, carbetocin displays a five‐fold right‐shifted EC_50_ value and a 50% reduction in the maximal OXTR/Gq activation value, consistent with a partial agonist profile. These results are in agreement with previous myometrial contractility studies performed *in vitro* on isolated rat uterine strips reporting an EC_50_ value of carbetocin (48 nm) approximately ten times higher than that of oxytocin (5.62 nm) and a maximal contractile responsiveness (E_max_) of carbetocin approximately 50% lower than that of oxytocin 34. Substitutions that modify the disulphide bond of oxytocin by introducing a dicarba bond were shown not only to significantly decrease the biological activity of the peptide, but also to induce a loss of receptor selectivity or a switch from an agonist to an antagonist profile 39. Because the crystal structure of carbetocin is currently unavailable, we can only speculate about the molecular basis of these peculiar agonistic properties. In particular, we hypothesise that the modification of the disulphide bond generates distortions in carbetocin secondary structure, inducing different interactions between its cyclic part and the cluster of residues located in the transmembrane helices TM3, TM5 and TM6 of OXTR. These interactions stabilise different receptor conformations that influence OXTR activation 40 and G‐protein coupling selectivity 41 and could be particularly relevant for determining the functional selectivity and partial agonistic properties of oxytocin analogues. Further experiments aiming to investigate the secondary structure of carbetocin, as well as modelling and mutagenesis studies, will be necessary to address this hypothesis further. The unique functional selective OXTR/Gq coupling of carbetocin could be particularly relevant in neuronal cells, where OXTR coupling to Gq and Gi/Go can result in opposite effects on cell excitability via inhibition or activation of potassium channels 42. In this context, carbetocin could be instrumental in identifying the role played by OXTR/Gq coupling with respect to eliciting specific behavioural and neuroendocrine effects, particularly if combined with DNalOVT and atosiban, respectively a Gi1 and a Gi3 functional selective agonists described previously 15. Such studies would provide the rationale for pharmacological treatments based on ‘functional selective agonists’ characterised by their specific G‐protein isoform coupling. We have previously demonstrated that oxytocin has a strong mitogenic effect when OXTRs are localised in plasma membrane domains enriched in caveolin proteins, glycosphingolipids and cholesterol (lipid rafts), whereas it inhibits cell growth when the receptors are excluded from these domains 43, 44. Moreover, we found that the inhibition of cell growth is mediated by a pertussis toxin‐sensitive G‐protein (Gi/o), whereas the stimulation of cell growth is mediated by a Gi/o‐independent pathway, likely via Gq activation 43, 44. Because carbetocin proved to be an OXTR/Gq functional selective agonist, it may have positive effects on cell proliferation. Experiments using this analogue are currently under way with the aim of testing this hypothesis.

It has been previously demonstrated that carbetocin binds not only to the OXTR, but also to vasopressin receptors 34. Competition binding studies performed on rat myometrial and kidney membranes using tritiated oxytocin and arginine vasopressin as radiotracers have reported K_i_ values of 1.96 nm for the rat OXTR, 7.24 nm for the rat V1 receptors (likely V1aR subtype) and 61.3 nm for the rat V2 receptor 34. Despite this previously reported high affinity binding, we did not observe any significant activation of hV1a and hV1b receptors with a concentration of carbetocin as high as 10 μm, suggesting that carbetocin can bind to these receptors without inducing any activation. Moreover, we observed that it can act as a competitive antagonist at the vasopressin receptors. This is an important feature that differentiates carbetocin from oxytocin because oxytocin is capable to bind to and activate both the V1a and V1b receptors 16, 35. Previously, a great deal of effort was devoted to the identification of OXTR selective antagonists and, more recently, interest in OXTR selective agonists has surged. At present, only one oxytocin peptide analogue, Thr4Gly7OT compound, has proved to be selective for the OXTR, even though its selectivity is limited to mice and rats because Thr4Gly7OT was demonstrated to be rather unselective towards the human oxytocin/V1 receptor subtypes 16, 45. Carbetocin, as a selective OXTR agonist potentially capable of blocking the vasopressin V1a and V1b receptors, represents an interesting agonist worthy of careful pharmacological characterisation *in vivo*, where it may elicit unexpected and presently not fully predictable effects.

Following oxytocin binding, we have previously shown that the OXTR recruited β‐arrestin and underwent a rapid (2–10 min) internalisation process followed by receptor recycling to the plasma membrane 36. We report here that, upon carbetocin exposure, the OXTR is efficiently internalised in the absence of any significant recruitment of either β‐arrestin1 or β‐arrestin2. Moreover, once internalised in response to carbetocin, the OXTR remains in the intracellular compartments and does not recycle back to the plasma membrane. Other GPCRs were shown to be internalised independently of the recruitment of β‐arrestins, such as the ghrelin receptor 46 and the serotonin 5HT‐2A receptor 47. One possible mechanism for such β‐arrestin‐independent internalisation relies on the direct binding of an adaptor protein, such as AP2, directly to the OXTR; however, the OXTR lacks the polyarginine motif that is required for direct AP2 interaction 37, making this possibility unlikely. GRK2, a kinase that has been demonstrated to interact with the OXTR 48 can also function as an adaptor protein by interacting directly with clathrin via a clathrin box, mediating a β‐arrestins‐independent internalisation process 49.

Interestingly, although β‐arrestins were not required for carbetocin‐induced OXTR internalisation, no OXTR recycling was observed in the absence of the recruitment of β‐arrestins. β‐arrestins are fundamental for regulating not only the internalisation, but also the recycling of a number of GPCRs. Recycling of GPCRs is considered to occur following endosome acidification, β‐arrestin dissociation and receptor dephosphorylation, a series of processes that occur as the receptor traffics through the vesicular compartments of the cytoplasm. Because arrestins serve as adaptors for many proteins, it is intriguing to speculate that they can specifically regulate the post‐endocytotic intracellular trafficking of GPCRs and/or the activity of phosphatases. In particular, it has been demonstrated that, in the absence of β‐arrestins, the N‐formyl peptide receptor undergoes ligand‐induced internalisation but is trafficked improperly within the cell, resulting in intracellular retention and recycling to the plasma membrane not being possible 50. Whatever the mechanism involved, it is relevant that carbetocin disclosed a β‐arrestin‐independent internalisation pathway of the OXTR, which is described here for the first time.

The peculiar effects of carbetocin on receptor recycling should be considered when repeated applications are intended to be used for therapeutic purposes because the absence of receptor recycling could lead to tolerance, a pharmacologically defined phenomenon that consists of a smaller response being observed after repeated exposure to a drug 51. Tolerance can be a result of a reduction in functional receptor at the plasma membrane, as in the case of an agonist of the motilin receptors, a drug used as a gastrointestinal prokinetic agent 52. In the case of octreotide, a somatostatin receptor (SST2R) agonist used for controlling hormone‐related symptoms of functioning gastroenteropancreatic neuroendocrine tumours, the antisecretory potency decreased with long‐term treatment as a consequence of the persistent internalisation of SST2R 53. Finally, a rebound effect can appear after the discontinuation of a drug 54. There are many classes of medications that produce rebound, including antidepressants, opioids and β‐adrenoreceptor blockers and, in most cases, the rebound effect is associated with an up‐regulation of receptors 54. Upon these considerations, variations in OXTR expression levels after carbetocin administration should be subject to careful future investigation when planning its use *in vivo*.

## Conclusions

The present study indicates that carbetocin and oxytocin display substantial differences in a number of key molecular pharmacological properties. On the one hand, these differences may lead to interesting new effects of carbetocin compared to oxytocin. On the other hand, caution should be used when employing carbetocin as a substitute for oxytocin because unexpected (and at present unpredictable) effects could be encountered, particularly *in vivo*. This is especially true for the use of carbetocin in neurodevelopmental and psychiatric conditions, in which the precise role of OXTR and V1 receptors is far from clear. Further translational studies are needed to clarify and fully exploit the therapeutic value of this analogue.
